# Optimal timing for intermittent administration of parathyroid hormone (1–34) for distraction osteogenesis in rabbits

**DOI:** 10.1186/s13018-022-03019-2

**Published:** 2022-03-03

**Authors:** Narisaku Inada, Tetsuya Ohata, Hideto Maruno, Takeshi Morii, Naobumi Hosogane, Shoichi Ichimura

**Affiliations:** Department of Orthopedic Surgery, School of Medicine, Kyorin University, 6-20-2 Shinkawa, Mitaka, Tokyo 181-8611 Japan

**Keywords:** Parathyroid hormone, Distraction osteogenesis, Callus strength, Bone mineral density, Administration timing

## Abstract

**Background:**

To date, the usefulness of parathyroid hormone [PTH (1–34)] in distraction osteogenesis has been reported in several studies. We aimed to determine the optimal timing of PTH (1–34) administration in a rabbit distraction osteogenesis model.

**Methods:**

The lower hind leg of a Japanese white rabbit was externally fixed, and tibial osteotomy was performed. One week after the osteotomy, bone lengthening was carried out at 0.375 mm/12 h for 2 weeks. After 5 weeks, the lower leg bone was collected. Bone mineral density (BMD), peripheral quantitative computed tomography (pQCT), micro-computed tomography (micro-CT), and mechanical tests were performed on the distracted callus. The rabbits were divided into three groups according to the timing of PTH (1–34) administration: 4 weeks during the distraction and consolidation phases (group D + C), 2 weeks of the distraction phase (group D), and the first 2 weeks of the consolidation phase (group C). A control group (group N) was administered saline for 4 weeks during the distraction and consolidation phases. Furthermore, to obtain histological findings, lower leg bones were collected from each rabbit at 2, 3, and 4 weeks after osteotomy, and tissue sections of the distracted callus were examined histologically.

**Results:**

The BMD was highest in group C and was significantly higher than group D. In pQCT, the total cross-sectional area was significantly higher in groups D + C, D, and C than group N, and the cortical bone area was highest in group C and was significantly higher than group D. In micro-CT, group C had the highest bone mass and number of trabeculae. Regarding the mechanical test, group C had the highest callus failure strength, and this value was significantly higher compared to group N. There was no significant difference between groups D and N. The histological findings revealed that the distracted callus mainly consisted of endochondral ossification in the distraction phase. In the consolidation phase, the chondrocytes were almost absent, and intramembranous ossification was the main type of ossification.

**Conclusion:**

We found that the optimal timing of PTH (1–34) administration is during the consolidation phase, which is mainly characterized by intramembranous ossification.

## Background

**Fig. 1:**
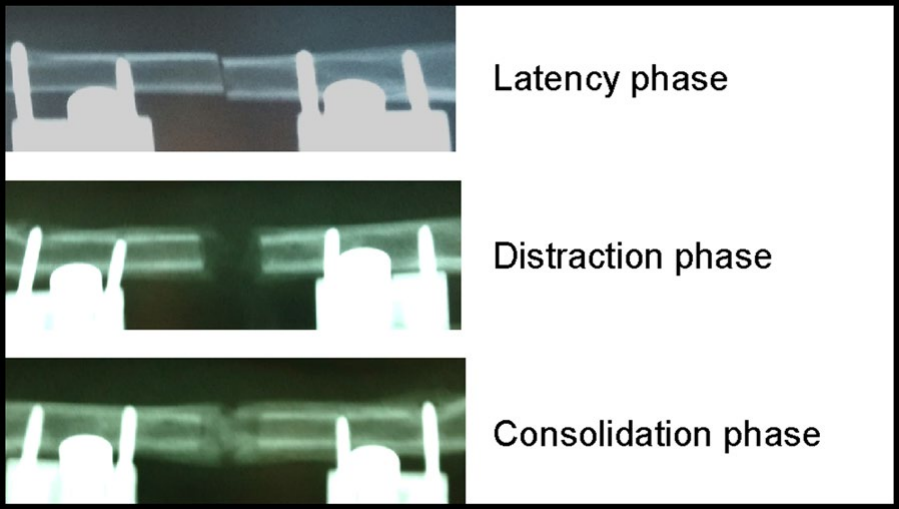
Three phases in distraction osteogenesis. There are three phases in the course of distraction osteogenesis (latency, distraction, and consolidation). The latency phase is the waiting period from the time of osteotomy to the start of distraction. The distraction phase is the period in which tension is applied to the callus at a constant rate and rhythm to extend it to the desired length. The consolidation phase is the period in which the callus undergoes mineralization and finally stabilizes

Distraction osteogenesis is a surgical technique that ensures new bone formation in the distraction gap by adding traction stress to the osteotomy site. This process has three phases: (1) latency phase—the waiting period from osteotomy to the start of distraction, (2) distraction phase—the period of bone lengthening, and (3) consolidation phase—the waiting period for callus maturation after the end of distraction [1–3] (Fig. 1). Distraction osteogenesis is used in clinical practice to treat various bone defects [4–10]. Other surgical techniques for bone defects include vascularized bone grafts [11] and the Masquelet technique [12–14]. Compared to these techniques, the advantage of distraction osteogenesis is that normal tissue is not sacrificed because bone grafting is unnecessary and special techniques such as microsurgery are not required. On the other hand, the disadvantage is the long period required for bone fusion, during which an external fixator must be worn for an extended period. Clinically, 1 cm of distracted callus takes 1 month to mature and heal [15]. In the case of a 10-cm bone defect, an external fixator should be worn for 10 months. Long-term external fixation may increase the burden on daily and social life, economic and mental burden, and risk of infection at the pin insertion site [16–18]. Therefore, the treatment period of distraction osteogenesis needs to be shortened. There are ongoing attempts to combine distraction osteogenesis with other treatment modalities, such as ultrasonic waves [19], extracorporeal shock waves [20], alternating current electrical stimulation [21], dynamization [22], and various bone metabolism regulators and cytokine administration [23–30]. The parathyroid hormone (PTH (1–34)) has been applied clinically to treat osteoporosis and has excellent efficacy in increasing bone mineral density (BMD) and inhibiting bone fractures [31]. The usefulness of PTH (1–34) in fracture healing has been widely studied. In a rat femoral fracture model, PTH (1–34) has been shown to increase BMD, ultimate load, callus volume, callus strength, and promote fracture healing [32–37]. A recent review article also showed that intermittent administration of PTH (1–34) promotes fracture healing in animal studies [38]. Human studies are few and inconsistent with animal studies, but they provide insight into the potential for intermittent PTH (1–34) administration to promote fracture healing. Another meta-analysis has shown that PTH treatment in patients with fracture was better than a placebo or no treatment based on the time for fracture healing, the degree of fracture pain, and the functional outcomes [39].

To date, the usefulness of PTH (1–34) in distraction osteogenesis has been noted only in a few reports [27–30]. Among them, Aleksyniene et al. [27] found that callus strength, callus volume, and BMD were not significantly different when PTH (1–34) was administered during the entire distraction and consolidation phases or when administered only during the consolidation phase in a rabbit distraction osteogenesis model. However, they did not compare the results with those of the group that received PTH (1–34) only in the distraction phase. No studies have evaluated the optimal timing of PTH (1–34) administration during the distraction and consolidation phases, considering that the type of ossification is different in these two phases [40, 41]. As the type of ossification may differ during different phases of distraction osteogenesis model, we have assumed the existence of an optimal timing of PTH (1–34). Therefore, this study aimed to elucidate the optimal timing of PTH (1–34) administration by biologically evaluating each phase.

## Methods

### Animals and test groups

Twenty-one-week-old Japanese male white rabbits (Oriental Yeast Co., Ltd., Tokyo, Japan) weighing 2.5–2.8 kg were used. In accordance with a previous study, PTH (1–34) was subcutaneously injected at a volume of 30 μg/kg every other day [30]. A total of 28 rabbits included in this study were divided into four groups (group D + C, D, C, N), each with seven rabbits. In group D + C, PTH (1–34) was administered for 4 weeks (2 weeks distraction phase + 2 weeks consolidation phase) from the start of distraction. In group D, PTH (1–34) was administered for 2 weeks during the distraction phase, and saline was administered for 2 weeks during the consolidation phase. In group C, saline was administered for 2 weeks during the distraction phase, and PTH (1–34) was administered for 2 weeks during the consolidation phase. Group N was the control, and saline was administered for 4 weeks from the start of distraction.

In addition, to confirm the type of ossification at the distraction site in this experimental model, a histological examination of the distracted callus was performed on the three Japanese male white rabbits that were administered with saline for 4 weeks from the start of distraction (same as group N) and three rabbits administered with PTH (1–34) also for 4 weeks (same as group D + C). The histological examination was performed at the distraction phase (1 week after the start of distraction), at the end of the distraction phase (2 weeks after the start of distraction), and at the consolidation phase (1 week after the end of distraction).

### Administration of PTH (1–34)

The PTH (1–34) used in this study was human PTH (1–34), teriparatide acetate (Asahi Kasei Pharma, Tokyo, Japan), which is a sequence of 34N-terminal amino acids. The molecular weight of teriparatide is 4418 Da. A 10 μg/mL PTH solution was prepared using saline as a solvent, and this was divided into 1-mL containers and cryopreserved at − 80 °C. The PTH solution was thawed at room temperature immediately before administration. The amount of solution corresponding to the body weight of each individual rabbit was calculated, and the required amount was subcutaneously injected. The same amount of saline was used in a control group.

### Distraction osteogenesis model

**Fig. 2:**
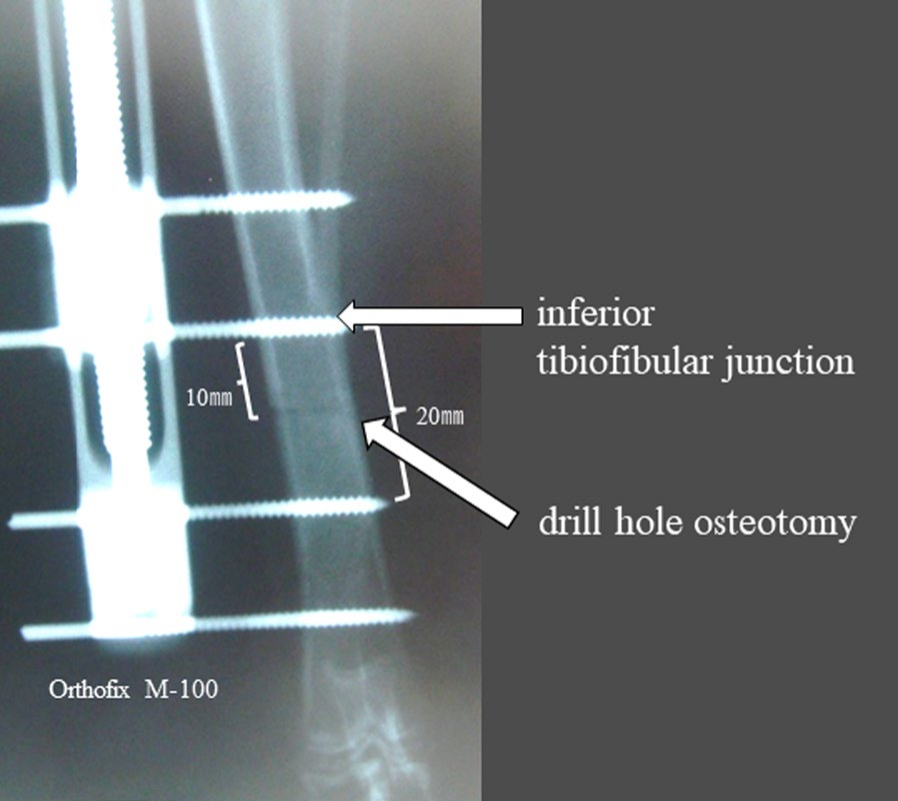
Position of osteotomy and pin insertion for the external fixator. The Orthofix M-100 was fixed to the tibia with four 2.0-mm half pins. The middle two pins were set 20 mm apart and drill hole osteotomy was performed between the two pins, i.e., 10 mm distal to the inferior tibiofibular junction

**Fig. 3:**
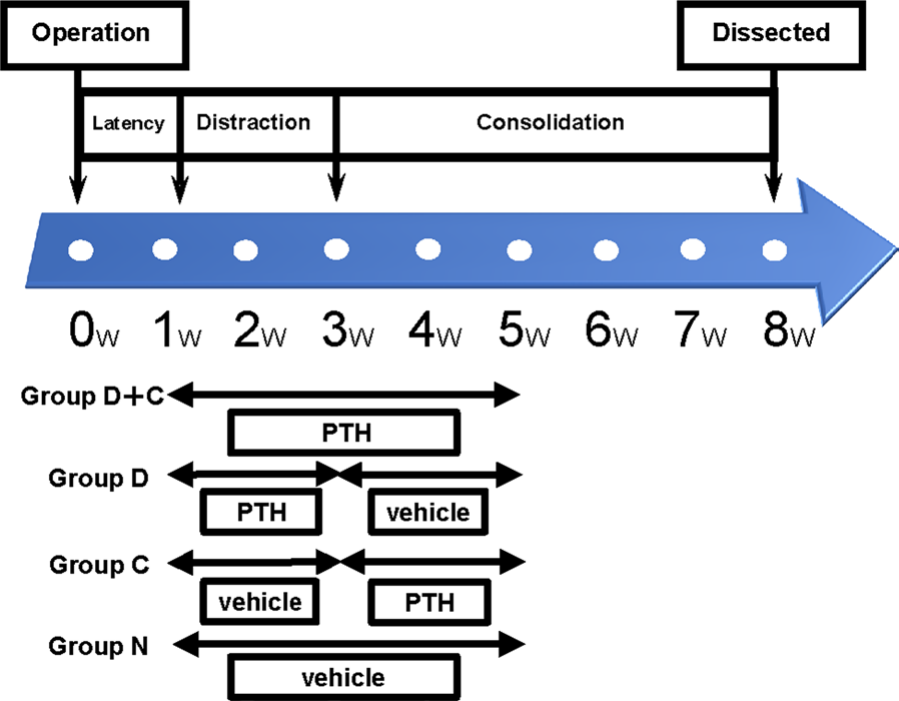
Experimental protocol. The 1-week latency phase was followed by a 2-week distraction phase and a 5-week consolidation phase. Group D + C received PTH (1–34) for 4 weeks from the start of distraction. Group D received PTH (1–34) for 2 weeks from the start of distraction and saline for the subsequent 2 weeks. Group C received saline from the start of distraction and PTH (1–34) for the subsequent 2 weeks, and group N received saline solution for 4 weeks from the start of distraction. The experimental animals were euthanized at 8 weeks after osteotomy

The distraction osteogenesis model was based on Maruno’s method [29]. For anesthesia, ketamine hydrochloride 15 mg/kg (Sankyo Pharmaceutical Co., Ltd., Tokyo, Japan) was intramuscularly injected, and then pentobarbital 30 mg/kg (Tanabe Pharmaceutical Co., Ltd., Tokyo, Japan) was intravenously administered. A skin incision of approximately 40 mm was made on the medial side of the lower right hind leg to expose the periosteum on the medial aspect of the tibia. Without peeling the periosteum, two half pins with a diameter of 2.0 mm were inserted across the inferior tibiofibular junction at an intermediate distance of 20 mm, and an external fixator for the short tubular bone of humans (Orthofix M-100; Verona, Italy) was attached. The distance between the two central pins was 20 mm, and the bone was cut at 10 mm distal to the inferior tibiofibular junction using a drill hole (Fig. 2). During osteotomy, saline was added dropwise to the osteotomy site to prevent heating. The skin was sutured after turning the extender in the direction for shortening to avoid the gap at the osteotomy site. The left hind leg was not treated. The hind legs were loaded immediately after surgery without any restriction. One week after the osteotomy, the bones were distracted by 0.375 mm every 12 h for two consecutive weeks, according to the method of Little et al. [26]. The amount of bone distraction was 0.75 mm per day, and the total distraction distance was 10.5 mm, corresponding to approximately 9.4% of the total tibial length. Following completion of the 2-week extension, the external fixator was removed after the waiting period of 4 weeks. Eight weeks after the operation, the rabbits were euthanized under deep anesthesia by an overdose of pentobarbital, and the lower leg bone was collected (Fig. 3).

### Measurement methods

The collected lower leg bone was wrapped in saline-soaked gauze and cryopreserved at − 80 °C until each test was performed. At the time of measurement, the tibia was naturally thawed at room temperature, and various imaging tests were performed, followed by a three-point bending test. The following parameters were measured and compared between groups.

#### Bone mineral density (BMD)

BMD of the distracted callus was measured by dual-energy X-ray absorptiometry (DXA) for small animals using DCS-600EX-R (Aloka, Japan). BMD was measured 4 mm both proximally and distally from the osteotomy site.

#### Peripheral quantitative computed tomography (pQCT)

BMD measuring device for small animals, XCT Research SA + pQCT (Stratec Medizintechnik GmbH, Pforzheim, Germany), was used to assess the cross-sectional shapes of the distracted callus. Measurement parameters included total (mm^3^) and cortical (mm^3^) areas of the distracted callus.

#### Micro-computed tomography (micro-CT)

Micro-CT was measured using a high-resolution microfocus X-ray CT scanner, ScanX-mate-A090S (Comscan Techno, Japan), to evaluate the three-dimensional morphology of the cortex and trabecular structure of the distracted callus. Bone volume/tissue volume (BV/TV), trabecular number (Tb.N), and trabecular separation (Tb.Sp) were measured as parameters of the micro-trabecular structure.

#### Mechanical testing (three-point bending test)

The three-point bending test was performed using GRAPH-2000E (Shimadzu, Japan). The anterior surface of the tibia faced upward, whereas the posterior surface faced downward to allow the center of the distraction osteogenesis to be located in the middle between two support points at 20 mm distance. Then, pressure was applied with a crosshead speed of 2 mm/s at the center of the distracted callus from the anterior tibia surface. The measurement parameter was absorption energy until callus fracture [work to failure (Nmm)].

### Histological examination

The lower leg bone collected at each evaluation period was decalcified using formic acid-formalin and was cut into sagittal sections, which were stained using hematoxylin and eosin.

### Statistical analysis

For statistical analysis, JMP® 15 (SAS Institute Inc., Cary, NC, USA) statistical software was used, with the significance set at *p* < 0.05. Values are expressed as mean ± standard deviation (SD). Differences between groups were tested by analysis of variance with a Tukey–Kramer honestly significant difference for post hoc analysis.

### Ethics

This study conformed to Japan’s “Law concerning the protection and control of animals,” “Standard concerning the breeding and protection of laboratory animals,” “Laboratory Animal Guidelines of Kyorin University,” and other relevant guidelines. The study was conducted at a laboratory of the Department of Orthopedic Surgery, Kyorin University.

## Results

Six rabbits were excluded due to loosening of the external fixator pin or fracture during the treatment course, and the remaining 22 rabbits (seven rabbits in group D + C, five rabbits in group D, five rabbits in group C, and five rabbits in group N) were evaluated.

### Imaging tests

#### BMD (mg/cm^2^) (Table 1)

**Table 1:** BMD measured with DXA, and total area and cortical area measured with pQCT

	*n*	BMD (mg/cm^2^)	Total area (mm^3^)	Cortical area (mm^3^)
Group D + C	7	206 ± 28	76 ± 2^#^	25 ± 3
Group D	5	196 ± 15	81 ± 3^#^	24 ± 1
Group C	5	256 ± 18*	76 ± 1^#^	28 ± 1*
Group N	5	239 ± 42	70 ± 1	25 ± 3

The values are presented as mean ± standard deviation

*p < 0.05 versus Group D

^#^*p* < 0.05 versus Group N

The BMD (mean ± SD) of the whole distracted callus by DXA was highest in group C (256 ± 18) and was significantly higher compared with group D (196 ± 15).

#### pQCT (Table 1)

The total area (mean ± SD) was highest in group D. Compared with group N, groups D + C, D, and C were significantly higher by 9%, 16%, and 9%, respectively. The cortical area (mean ± SD) was highest in group C and was significantly different compared with group D.

#### Micro-CT (Table 2)

**Table 2:** Micro-architecture of the distracted callus evaluated using micro-CT

	*n*	BV/TV (%)	Tb.N (1/mm)	Tb.Sp (μm)
Group D + C	7	44 ± 14	3.0 ± 0.8	235 ± 99
Group D	5	29 ± 4	2.1 ± 0.6	383 ± 156
Group C	5	49 ± 3*	3.2 ± 0.4*	172 ± 29*
Group N	5	49 ± 13	2.8 ± 0.2	229 ± 83

The values are presented as mean ± standard deviation

*p < 0.05 versus Group D

**Fig. 4:**
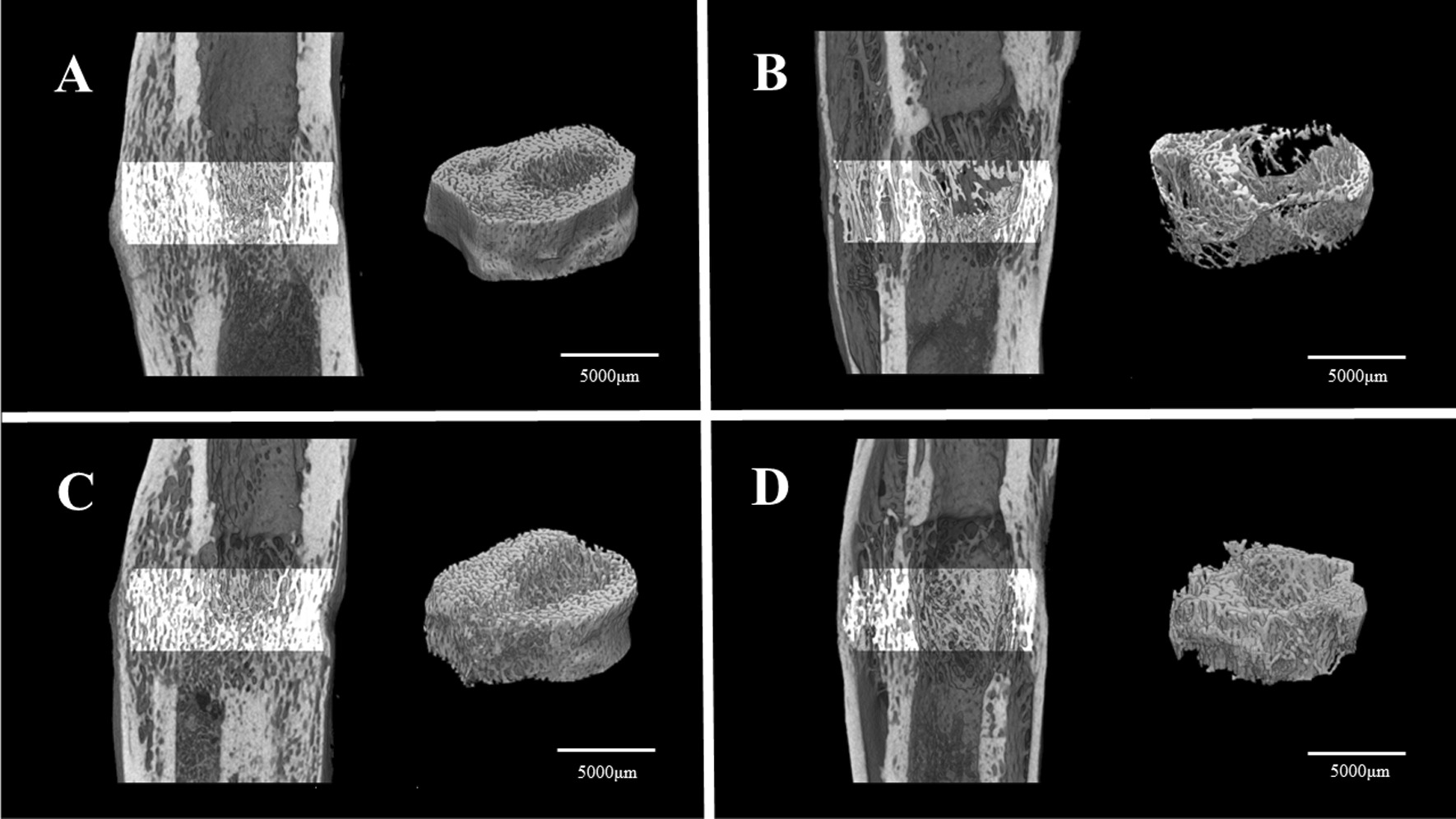
Three-dimensional micro-computed tomography images of the distracted callus. Longitudinal (left side) and transverse (right side) sections for a representative specimen. **A** Group D + C; **B** Group D; **C** Group C; **D** Group N

Three-dimensional images of the trabecular bone evaluated with micro-CT are shown in the figure (Fig. 4). The bone marrow space of A (group D + C) and C (group C) is filled with numerous trabeculae and low porosity, whereas B (group D) and D (group N) have fewer and thinner trabeculae in the marrow space. The architectural differences are quantified in Table 2. BV/TV (mean ± SD) and Tb.N (mean ± SD) were both highest in group C, and there were significant differences compared with group D. Tb.Sp (mean ± SD) was lowest in group C and showed a significant difference to group D.

### Mechanical testing (three-point bending test) (Fig. 5)

**Fig. 5:**
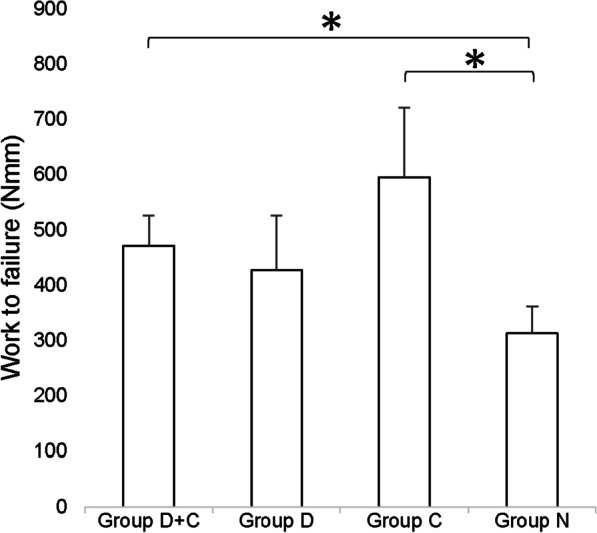
Mechanical properties by three-point bending analysis. The measurement parameter was absorption energy until the distracted callus fracture. The work to failure (mean ± SD) was highest in group C. Compared with group N, the work to failure for groups C and D + C increased by 90% and 50%, respectively, with statistical significance. There was no significant difference between groups D and N and between groups C and D + C. *p < 0.05, among four groups

The work to failure (mean ± SD) was 471 ± 55 in group D + C, 427 ± 99 in group D, 595 ± 126 in group C, and 313 ± 48 in group N. The work to failure (mean ± SD) was highest in group C. Compared with group N, the work to failure for groups C and D + C increased by 90% and 50%, respectively, with statistical significance. There was no significant difference between groups D and N and between groups C and D + C.

### Histological examination (Fig. 6)

**Fig. 6:**
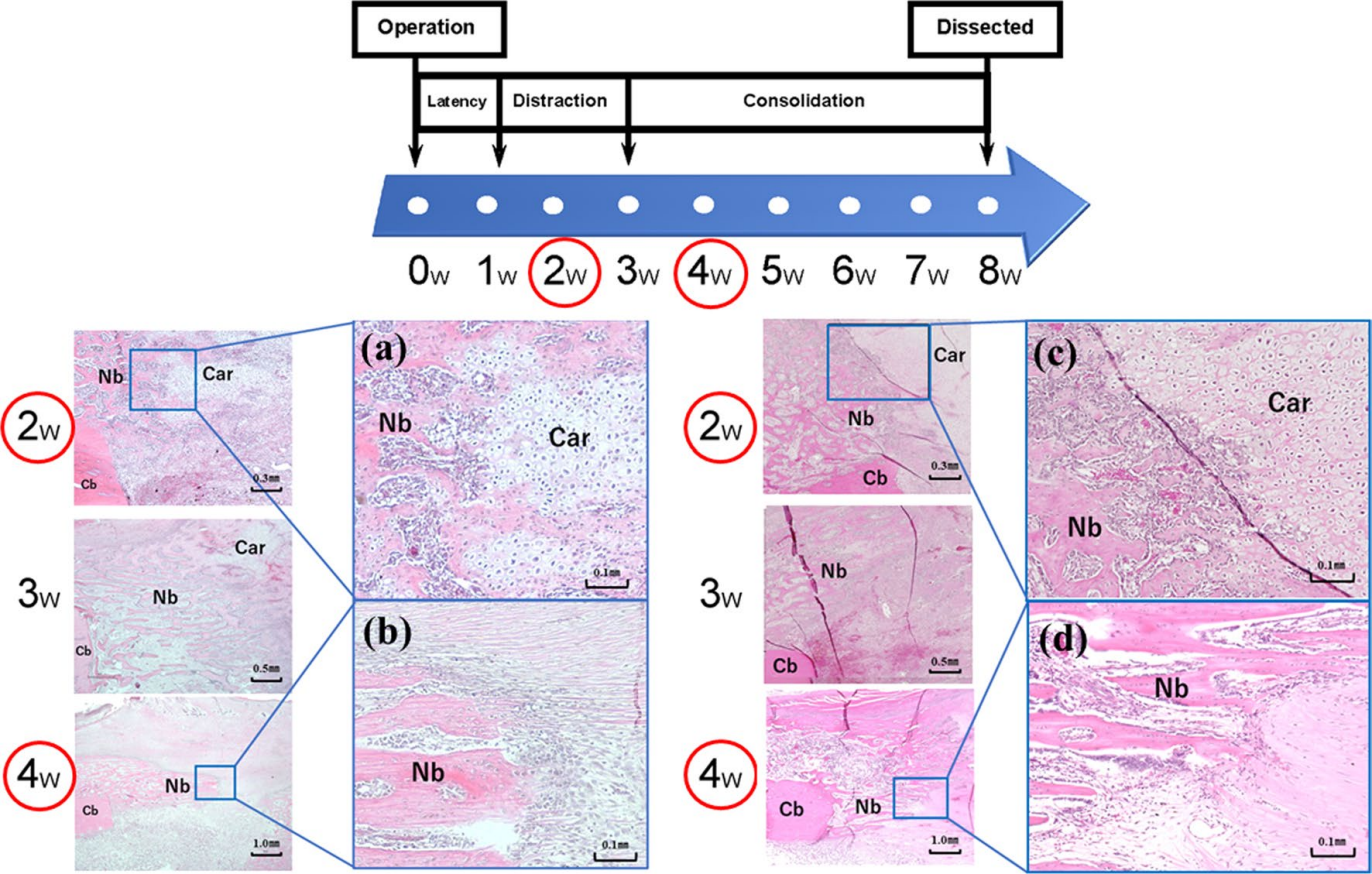
Histological analysis. Representative histological sections at the distracted callus stained with hematoxylin and eosin. a The histology of the distraction phase by the protocol in group N. b The histology of the consolidation phase by the protocol in group N. c The histology of the distraction phase by the protocol in group D + C. d The histology of the consolidation phase by the protocol in group D + C. Each of histological images a–d was high-power fields of the area around the distal part of the osteotomy where new bone is beginning to appear. Chondrocytes were abundant in the distracted callus 2 weeks after the osteotomy (distraction phase), and a new bone was formed by endochondral ossification. The woven bone with some cartilage is predominant. Almost no chondrocytes were observed in the distracted callus 4 weeks after osteotomy (consolidation phase), and a new bone was formed by intramembranous ossification. The mature lamellar bone is predominant. *Car* cartilage cell, *Nb* new bone, *Cb* cortical bone, *2w* 2 weeks after the osteotomy, *3w* 3 weeks after the osteotomy, *4w* 4 weeks after the osteotomy

The type of ossification of new bone that has begun to appear around the distal part of the osteotomy was shown. The images on the left showed the histology of the distraction phase (Fig. 6a) and the consolidation phase (Fig. 6b) by the protocol in group N. The images on the right showed the histology of the distraction phase (Fig. 6c) and the consolidation phase (Fig. 6d) by the protocol in group D + C. In this case, the distraction phase represented 2 weeks after the osteotomy (1 week after the start of distraction) and the consolidation phase represented 4 weeks after the osteotomy (1 week after the end of distraction). A different type of ossification was seen between the distraction and consolidation phases in both group N and group D + C. At the distraction phase, the chondrocytes were abundant in the distracted callus, and the new bone was formed by endochondral ossification (Fig. 6a, c). The ossification occurred through a cartilage intermediate. At the end of the distraction phase, 2 weeks after the start of distraction, the chondrocytes decreased and intramembranous ossification was notable. At the consolidation phase, only a few chondrocytes were identified, and the new bone in the callus distraction part was almost formed by intramembranous ossification. It seemed that there was a difference in the thickness and quantity of the new bone between the protocol in group N and D + C (Fig. 6b, d). There was no qualitative difference between the N and D + C groups; however, they were observed to be somewhat different quantitatively.

## Discussion

Intermittent administration of PTH (1–34) promotes the differentiation of mesenchymal stem cells into osteoblasts and accelerates bone formation. Studies using ovariectomized cynomolgus monkeys [42–44] have shown that PTH (1–34) administration significantly increases bone mass, improves bone micro-architecture, and increases bone strength in the vertebral body and femoral neck. Furthermore, in a study using human iliac bone biopsy specimens, PTH (1–34) administration showed an increase in total bone mass. Significant improvements in the trabecular structure model index, trabecular connectivity density, and cortical bone width were observed compared to the placebo group [45]. However, the mechanism by which PTH (1–34) activates bone metabolism predominantly in bone formation remains largely unknown.

To date, research on the usefulness of PTH (1–34) in distraction osteogenesis has been limited. Seebach et al. used a rat femur extension model with PTH (1–34) administration, 60 μg/kg daily, for a 10-day distraction phase and a 20-day consolidation phase. Following the study, they reported a 58% increase in callus mass, 24% increase in BMD, and 50% increase in maximum point load [28]. Aleksyniene et al. [27] used a rabbit tibial distraction model with PTH (1–34) administration, 25 μg/kg daily, for a 10-day distraction phase and a 20-day consolidation phase, and they reported an increase in BMD and bone strength. Recently, by using a rabbit distraction osteogenesis model, we reported an increase in the amount of cortical bone formation and callus width, as well as a significant increase in callus strength after administrating 30 μg/kg of PTH (1–34) every other day over a 14-day distraction phase and 35-day consolidation phase [29]. In addition, we have shown that PTH (1–34) administration shortened the callus maturation period by 2 weeks [30]. Although the PTH (1–34) dose, administration interval, and duration of the distraction and consolidation phases used in our study were different to the study reported by Aleksyniene et al., both emphasized the usefulness of PTH (1–34) for rabbit distraction osteogenesis.

Regarding the optimal timing of PTH (1–34) administration for distraction osteogenesis, Aleksyniene et al. [27] reported that administrating PTH (1–34) for both distraction and consolidation phases was not superior to PTH (1–34) at only the consolidation phase in the callus volume, BMD, and work to failure. Richards et al. [46] investigated the bone formation in a rabbit tibial distraction osteogenesis model and showed that bone formation was the best in the first half of the consolidation phase, suggesting that this phase may be the optimal time for biological and mechanical interventions for promoting bone regeneration. In a recent distraction osteogenesis study in humans, Wagner et al. reported that administrating PTH (1–34) during the consolidation phase raised the rate of regenerated calcification twofold compared to patients without PTH (1–34) [47]. In our current study, for the first time, PTH (1–34) was administered separately during the distraction and consolidation phases to elucidate the optimal administration timing. Our results showed that BMD, cortical area, and the distracted callus strength were highest in the group that received PTH (1–34) in the consolidation phase only. In addition, the total distracted callus area was significantly higher in all groups that received PTH (1–34) than in the control group regardless of the timing of administration. Except for the group that received PTH (1–34) during the distraction phase only, the distracted callus strength was significantly higher in the group that received PTH (1–34) than in the control group. The increase in the total cross-sectional area of callus was considered to be related to callus strength. However, although the total distracted callus area was highest in the group that received PTH (1–34) in the distraction phase only, the distracted callus strength was not significantly different compared with the control group. This result might have been influenced by the micro-architecture of the distracted callus. In general, cylindrical structures, such as long bones with a larger diameter and longer outer peripheral length, are known to have higher strength on the premise that the amount of the constituent materials is the same [48]. In micro-CT, the distracted callus micro-architecture of the group that received PTH (1–34) in the distraction phase only showed less bone mass and Tb.N and larger Tb.Sp. Therefore, the distracted callus strength was not sufficiently obtained, despite the large total area. In contrast, in the group that received PTH (1–34) during the consolidation phase only, the total distracted callus area was significantly larger than in the control group with higher bone mass and Tb.N and smaller Tb.Sp, which may lead to the highest strength.

In distraction osteogenesis, a new bone is predominantly formed by endochondral ossification in the distraction phase and by intramembranous ossification in the consolidation phase [40, 41]. However, the ossification form may vary according to the type of experimental animal and protocol. Therefore, the histological evaluations of the distracted callus were also confirmed in this study. Our results were consistent with previous reports, by endochondral ossification being predominant in the distraction phase and intramembranous ossification in the consolidation phase. The callus formed during the healing process after a fracture consists of a mixture of intramembranous and endochondral ossification [49], which is difficult to distinguish with histological examination. In contrast, in the distraction osteogenesis model, type of ossification is reproducible with the phase, such as distraction or consolidation phases. Based on our results that the consolidation phase is the optimal PTH (1–34) administration period, we believe that PTH (1–34) may play a dominant role in intramembranous ossification.

Furthermore, in fracture healing, PTH has been shown to promote the proliferation and differentiation of chondrocytes [50]. Therefore, even in distraction osteogenesis, PTH may also have a promoting effect on chondrocytes during the distraction phase. However, our results do not sufficiently support this conjecture. Accordingly, it is important to consider the difference in chondrogenesis between fracture repair and distraction osteogenesis. In fracture healing, large hematomas, which contain abundant mesenchymal cells and respond to PTH, form immediately after fracture, and endochondral bone formation robustly occurs in the first period. On the other hand, in distraction osteogenesis, due to fewer hematomas being present, substantially less cartilage is formed, and its formation is restricted to the early distraction phase. After this phase, it is rapidly resorbed [51]. Therefore, the effect of PTH (1–34) on endochondral ossification during the distraction phase may be difficult to fully achieve in distraction osteogenesis. In the present study, although BMD and the parameters evaluated with mechanical testing and micro-CT were slightly better in group C than group D + C (Fig. 5, Tables 1, 2), there were no statistically significant differences between the two groups indicating that PTH (1–34) administration did not have positive effect during the distraction phase as in group D. Later, during the consolidation phase, proliferating osteoprogenitor cells differentiate from osteoblasts into osteocytes, resulting in intramembranous ossification. We consider that PTH (1–34) may have its most significant effect on promoting the differentiation of osteoprogenitor cells during the consolidation phase. The histological results of this study showed that there seemed to be a difference in the thickness and quantity of the new bone in the consolidation phase between the N and D + C protocols (Fig. 6b, d). We were unable to confirm that this is due to PTH (1–34) administration because only one rabbit was examined at each time, as the main purpose of this histological examination was to verify that the type of ossification (endochondral or intramembranous) at the distraction site was reproduced in our experimental protocol, similar to the previous studies. In future research, quantitative evaluation of the multiple regenerated tissues at different phases using immunohistochemistry or other methods should be considered.

Despite the usefulness of PTH (1–34) for distraction osteogenesis, its application in clinical practice remains a challenge. PTH (1–34) is an expensive drug with a limited lifetime use of 2 years [52, 53]. Identification of the optimal time when PTH (1–34) acts most effectively on callus formation for distraction osteogenesis in humans will improve patient compliance and cost-effectiveness. Regardless of whether PTH (1–34) was administered during the distraction and consolidation phases or only during the consolidation phase, there was no significant difference in the mechanical and structural properties of the distracted callus. The current findings suggest a clinical implication that will definitely contribute to reduce the PTH (1–34) treatment time in distraction osteogenesis.

This study had several limitations. First, in this work, PTH (1–34) was administered only in the first half of the consolidation phase, the effect of PTH (1–34) administration in the latter half of the consolidation phase remains unknown and should be investigated in the future. Second, considering that the bone anabolic effect on PTH (1–34) differs depending on the administration method [54, 55], the optimal administration approach, including the administration interval and injection site, should be examined in the future. Third, the dose of PTH (1–34) used in this experiment was 30 μg/kg, which is equivalent to the dose of other similar animal experiments (25–60 μg/kg). However, this dose is much higher than that approved by the US Food and Drug Administration for the treatment of osteoporosis patients (0.25–0.5 μg/kg/day). The dosage of PTH (1–34) needs to be investigated further for clinical application in humans. Fourth, criticism may be raised that the effect of PTH (1–34) on the contralateral tibia was not confirmed in the present this study. In the previous study, however, the effect of PTH (1–34) was analyzed in the intact contralateral tibia, and to a lesser extent than that observed in the distracted tibia [27]. In addition, PTH (1–34) was reported to have a stronger effect on bone with an activated repair response than on bone undergoing normal remodeling [28, 56]. Therefore, it would be expected to show a limited effect in the contralateral intact bone in our experiments as well; however, the intact bone was not taken in this study as our main purpose was to evaluate the effect of PTH (1–34) on distraction osteogenesis.

## Conclusions

In the rabbit distraction osteogenesis model, administration of PTH (1–34) during the consolidation phase formed a distracted callus with a stronger and better micro-architecture than the distraction phase. Furthermore, PTH (1–34) was shown to act more effectively for the intramembranous ossification during the consolidation phase than for the endochondral ossification during the distraction phase. Determining the optimal timing of PTH (1–34) administration may have a beneficial impact on patient compliance and cost-effectiveness.

## Data Availability

The datasets used and analyzed during the current study are available from the corresponding author on reasonable request.

## Abbreviations

BMD: Bone mineral density
BV/TV: Bone volume/tissue volume
DXA: Dual-energy X-ray absorptiometry
micro-CT: Micro-computed tomography
pQCT: Peripheral quantitative computed tomography
PTH: Parathyroid hormone
SD: Standard deviation
Tb.N: Trabecular number
Tb.Sp: Trabecular separation

## References

[1] Codivilla A (1994). On the means of lengthening, in the lower limbs, the muscles and tissues which are shortened through deformity. Clin Orthop Relat Res.

[2] Ilizarov GA (1989). The tension-stress effect on the genesis and growth of tissues. Part I. The influence of stability of fixation and soft-tissue preservation. Clin Orthop Relat Res.

[3] Ilizarov GA (1989). The tension-stress effect on the genesis and growth of tissues: part II. The influence of the rate and frequency of distraction. Clin Orthop Relat Res.

[4] Ilizarov GA (2006). The tension-stress effect on the genesis and growth of tissues. Clin Orthop Relat Res.

[5] Paley D, Maar DC (2000). Ilizarov bone transport treatment for tibial defects. J Orthop Trauma.

[6] Tsuchiya H, Tomita K, Minematsu K, Mori Y, Asada N, Kitano S (1997). Limb salvage using distraction osteogenesis: a classification of the technique. J Bone Joint Surg Br.

[7] Moseley CF (1989). Leg lengthening. A review of 30 years. Clin Orthop Relat Res.

[8] Paley D (1990). Problems, obstacles, and complications of limb lengthening by the Ilizarov technique. Clin Orthop Relat Res.

[9] Ilizarov GA (1990). Clinical application of the tension-stress effect for limb lengthening. Clin Orthop Relat Res.

[10] Aronson J (1997). Limb-lengthening, skeletal reconstruction, and bone transport with the Ilizarov method. J Bone Joint Surg Am.

[11] Malizos KN (2004). Free vascularized fibular grafts for reconstruction of skeletal defects. J Am Acad Orthop Surg.

[12] Masquelet AC, Begue T (2010). The concept of induced membrane for reconstruction of long bone defects. Orthop Clin N Am.

[13] Masquelet AC (2003). Muscle reconstruction in reconstructive surgery: soft tissue repair and long bone reconstruction. Langenbecks Arch Surg.

[14] Aho OM, Lehenkari P, Ristiniemi J, Lehtonen S, Risteli J, Leskelä HV (2013). The mechanism of action of induced membranes in bone repair. J Bone Joint Surg Am.

[15] Maffuli N, Fixsen JA (1996). Distraction osteogenesis in congenital limb length discrepancy: a review. J R Coll Surg Edinb.

[16] Makhdom AM, Hamdy RC (2013). The role of growth factors on acceleration of bone regeneration during distraction osteogenesis. Tissue Eng Part B Rev.

[17] Spiegelberg B, Parratt T, Dheerendra SK, Khan WS, Jennings R, Marsh DR (2010). Ilizarov principles of deformity correction. Ann R Coll Surg Engl.

[18] Iacobellis C, Berizzi A, Aldegheri R (2010). Bone transport using the Ilizarov method: a review of complications in 100 consecutive cases. Strat Trauma Limb Reconstr.

[19] Shimazaki A, Inui K, Azuma Y, Nishimura N, Yamano Y (2000). Low-intensity pulsed ultrasound accelerates bone maturation in distraction osteogenesis in rabbits. J Bone Joint Surg Br.

[20] Xiao-yu Y, Guang Y, Liang C, Ming C, Xiang-guo C, Yi-min C (2003). The effect of low energy extracorporeal shock wave on the callus formation during bone lengthening. J Jpn Assoc Extern Fixat Limb Lengthening.

[21] Kawamoto K, Kim W, Tsuchida Y, Tsuji Y, Fujioka M, Horii M (2005). Effect of alternating current electrical stimulation on lengthened callus. J Pediatr Orthop B.

[22] Kassis B, Glorion C, Tabib W, Blanchard O, Pouliquen JC (1996). Callus response to micromovement after elongation in the rabbit. J Pediatr Orthop.

[23] Okazaki H, Kurokawa T, Nakamura K, Matsushita T, Mamada K, Kawaguchi H (1999). Stimulation of bone formation by recombinant fibroblast growth factor-2 in callotasis bone lengthening of rabbits. Calcif Tissue Int.

[24] Schumacher B, Albrechtsen J, Keller J, Flyvbjerg A, Hvid I (1996). Periosteal insulin–like growth factor I and bone formation. Acta Orthop Scand.

[25] Tsubota S, Tsuchiya H, Shinokawa Y, Tomita K, Minato H (1999). Transplantation of osteoblast-like cells to the distracted callus in rabbits. J Bone Joint Surg Br.

[26] Little DG, Cornell MS, Hile MS, Briody J, Cowell CT, Bilston L (2001). Effect of pamidronate on distraction osteogenesis and fixator-related osteoporosis. Injury.

[27] Aleksyniene R, Thomsen JS, Eckardt H, Bundgaard KG, Lind M, Hvid I (2009). Three-dimensional microstructural properties of regenerated mineralizing tissue after PTH (1–34) treatment in a rabbit tibial lengthening model. J Musculoskelet Neuronal Interact.

[28] Seebach C, Skripitz R, Andreassen TT, Aspenberg P (2004). Intermittent parathyroid hormone (1–34) enhances mechanical strength and density of new bone after distraction osteogenesis in rats. J Orthop Res.

[29] Maruno H (2011). Effects of intermittent administration of parathyroid hormone on distraction osteogenesis in rabbits. J Kyorin Med Soc.

[30] Ohata T, Maruno H, Ichimura S (2015). Changes over time in callus formation caused by intermittently administering PTH in rabbit distraction osteogenesis models. J Orthop Surg Res.

[31] Neer RM, Arnaud CD, Zanchetta JR, Prince R, Gaich GA, Reginster JY (2001). Effect of parathyroid hormone (1–34) on fracture and bone mineral density in postmenopausal women with osteoporosis. N Engl J Med.

[32] Komatsubara S, Mori S, Mashiba T, Nonaka K, Seki A, Akiyama T (2005). Human parathyroid hormone (1–34) accelerates the fracture healing process of woven to lamellar bone replacement and new cortical shell formation in rat femora. Bone.

[33] Andreassen TT, Ejersted C, Oxlund H (1999). Intermittent parathyroid hormone (1–34) treatment increases callus formation and mechanical strength of healing rat fractures. J Bone Miner Res.

[34] Andreassen TT, Willick GE, Morley P, Whitfield JF (2004). Treatment with parathyroid hormone hPTH(1–34), hPTH(1–31), and monocyclic hPTH(1–31) enhances fracture strength and callus amount after withdrawal fracture strength and callus mechanical quality continue to increase. Calcif Tissue Int.

[35] Andreassen TT, Fledelius C, Ejersted C, Oxlund H (2001). Increases in callus formation and mechanical strength of healing fractures in old rats treated with parathyroid hormone. Acta Orthop Scand.

[36] Nakazawa T, Nakajima A, Shiomi K, Moriya H, Einhorn TA, Yamazaki M (2005). Effects of low-dose, intermittent treatment with recombinant human parathyroid hormone (1–34) on chondrogenesis in a model of experimental fracture healing. Bone.

[37] Nakajima A, Shimoji N, Shiomi K, Shimizu S, Moriya H, Einhorn TA (2002). Mechanisms for the enhancement of fracture healing in rats treated with intermittent low-dose human parathyroid hormone (1–34). J Bone Miner Res.

[38] Yamashita J, McCauley LK (2019). Effects of intermittent administration of parathyroid hormone and parathyroid hormone-related protein on fracture healing: a narrative review of animal and human studies. JBMR Plus.

[39] Hong H, Song T, Liu Y, Li J, Jiang Q, Song Q, Deng Z (2019). The effectiveness and safety of parathyroid hormone in fracture healing: a meta-analysis. Clinics (Sao Paulo).

[40] Kojimoto HA, Yasui NA, Goto TA, Matsuda SH, Shimomura YU (1988). Bone lengthening in rabbits by callus distraction. The role of periosteum and endosteum. J Bone Joint Surg Br.

[41] Yasui N, Sato M, Ochi T, Kimura T, Kawahata H, Kitamura Y (1997). Three modes of ossification during distraction osteogenesis in the rat. J Bone Joint Surg Br.

[42] Burr DB, Hirano T, Turner CH, Hotchkiss C, Brommage R, Hock JM (2001). Intermittently administered human parathyroid hormone(1–34) treatment increases intracortical bone turnover and porosity without reducing bone strength in the humerus of ovariectomized cynomolgus monkeys. J Bone Miner Res.

[43] Sato M, Westmore M, Ma YL, Schmidt A, Zeng QQ, Glass EV (2004). Teriparatide [PTH(1–34)] strengthens the proximal femur of ovariectomized nonhuman primates despite increasing porosity. J Bone Miner Res.

[44] Jerome CP, Burr DB, Van Bibber T, Hock JM, Brommage R (2001). Treatment with human parathyroid hormone (1–34) for 18 months increases cancellous bone volume and improves trabecular architecture in ovariectomized cynomolgus monkeys (*Macaca fascicularis*). Bone.

[45] Jiang Y, Zhao JJ, Mitlak BH, Wang O, Genant HK, Eriksen EF (2003). Recombinant human parathyroid hormone (1–34) [teriparatide] improves both cortical and cancellous bone structure. J Bone Miner Res.

[46] Richards M, Goulet JA, Schaffler MB, Goldstein SA (1999). Temporal and spatial characterization of regenerate bone in the lengthened rabbit tibia. J Bone Miner Res.

[47] Wagner F, Vach W, Augat P, Varady PA, Panzer S, Keiser S (2019). Daily subcutaneous teriparatide injection increased bone mineral density of newly formed bone after tibia distraction osteogenesis, a randomized study. Injury.

[48] Seeman E (2003). Periosteal bone formation a neglected determinant of bone strength. N Engl J Med.

[49] Bonnarens F, Einhorn TA (1984). Production of a standard closed fracture in laboratory animal bone. J Orthop Res.

[50] Ellegaard M, Jorgensen NR, Schwarz P (2010). Parathyroid hormone and bone healing. Calcif Tissue Int.

[51] Ai-Aql ZS, Alagl AS, Graves DT, Gerstenfeld LC, Einhorn TA (2007). Molecular mechanisms controlling bone formation during fracture healing and distraction osteogenesis. J Dent Res.

[52] Mori T, Crandall CJ, Ganz DA (2019). Cost-effectiveness of sequential teriparatide/alendronate versus alendronate-alone strategies in high-risk osteoporotic women in the US: analyzing the impact of generic/biosimilar teriparatide. JBMR Plus.

[53] Tashjian AH, Gagel RF (2006). Teriparatide [human PTH (1–34)]: 2.5 years of experience on the use and safety of the drug for the treatment of osteoporosis. J Bone Miner Res.

[54] Iida-Klein A, Zhou H, Lu SS, Levine LR, Ducayen-Knowles M, Dempster DW (2002). Anabolic action of parathyroid hormone is skeletal site specific at the tissue and cellular levels in mice. J Bone Miner Res.

[55] Zhou H, Iida-Klein A, Lu SS, Ducayen-Knowles M, Levine LR, Dempster DW (2003). Anabolic action of parathyroid hormone on cortical and cancellous bone differs between axial and appendicular skeletal sites in mice. Bone.

[56] Skripitz R, Andreassen TT, Aspenberg P (2000). Strong effect of PTH (1–34) on regenerating bone: a time sequence study in rats. Acta Orthop Scand.

